# A late and difficult diagnosis of ochronosis

**Published:** 2010-11-25

**Authors:** L Groseanu, R Marinescu, D Laptoiun, I Botezatu, F Staniceanu, S Zurac, R Ionescu

**Affiliations:** * Internal Medicine and Rheumatology Clinic, ‘St. Mary’ Clinical Hospital,‘Carol Davila’ University of Medicine and Pharmacy, Bucharest Romania; ** Orthopedics Clinic, Gh.N. Lupu Hospital, BucharestRomania

**Keywords:** ochronosis, alkaptonuria, discs calcification

## Abstract

Alkaptonuria is a rare autosomal recessive disorder of metabolism caused by deficiency of homogentisic acid oxidase and resulting in accumulation of homogentisic acid in collagenous structures. This causes the classic clinical triad: (1) homogentisic aciduria (urine blackens on standing when oxidized or alkalinized); (2)  eumelanin–like pigmentation of skin, sclera, cartilages, etc and (3) degenerative ochronic arthropathies usually in the fourth decade of life. Other important but more rare consequences of alkaptonuric ochronosis are cardiovascular and urinary tract involvement . We present a case of ochronosis with multiple visceral involvement : skin (fingers, ear sclera), severe spondylarthropaty with extensive calcifications of intervertebral discs and reduced mobility, osteoarthritis of both knees , right hip ostonecrosis , cardiovascular involvement ( severe  stenosis and  insufficiency of aortic valve that ) and urinary tract involvement (nephrolitiasis)

## Case report

A 55 year–old man was refered to our clinic in April 2008  for persistent right hip and low back pain not responding to NSAIDs and physiotherapy. Family history: mother diagnosed with ankylosing spondilitis. Personal history: 30 years prior to the presentation in our clinic he was diagnosed with ankylosing spondilitis, in 2002 he had surgery for kidney stones and he was diagnosed with arterial hypertension. 

**Figure 1:**
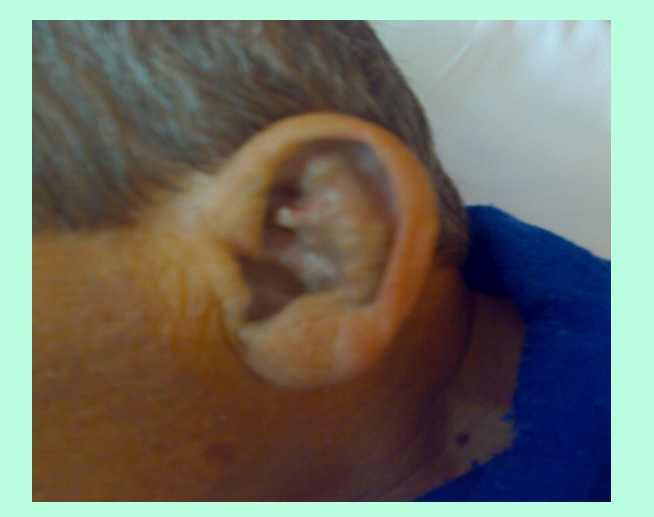


**Figure 2:**
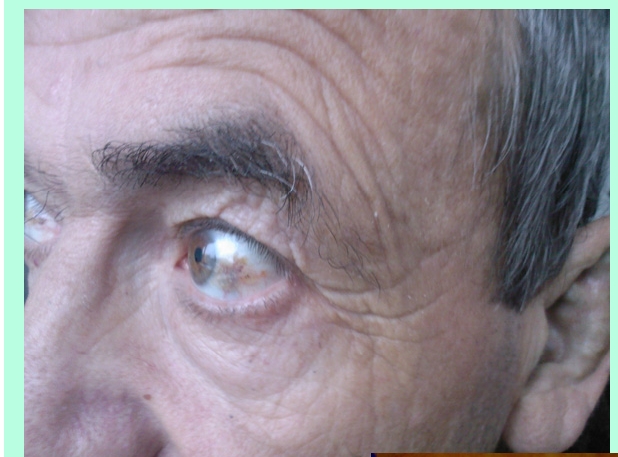


**Figure 3:**
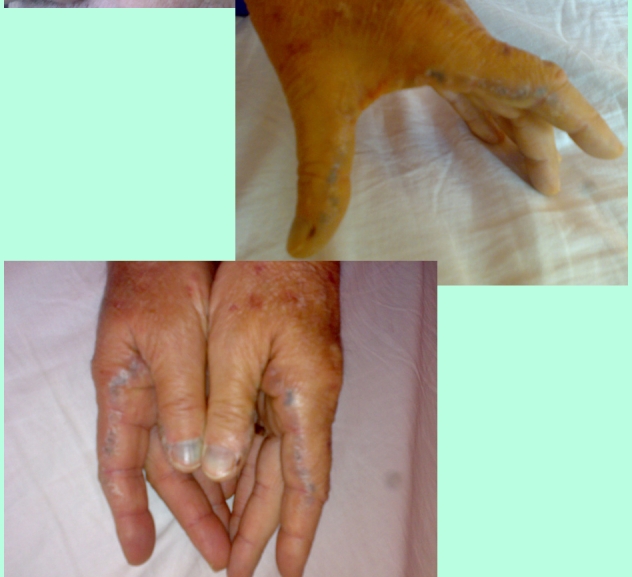


**Physical examination** revealed bluish–grey pigmentation of pinna ,thickened ear cartilage with  some visible calcifications  (Figure 1), the temporal region of scleras had grey–brown pigmentations (Figure 2) ; on the lateral side of the fingers black tattoo–like pigmentations were visible (Figure 3)

He had limited mobility of the spine: Schober=2.5cm, fingers–to–floor  index=22cm, occiput–to–wall index=8cm, chest expansion=2.5cm; loss of lombar lordosis, thoracic kyphosis,  inability to extend the neck. The hip mobility was very limited, he experienced difficulty in walking for a long time, the knees had a decreased range of motion  with crepitus , a valgum deformity was visible. Vital signs : BP=150/90mmHg, HR=82b/min, systolic bruit grade 3/6 at the base of the heart . 

**Laboratory findings**: ESR, CRP normal, hypercholesterolemia (300mg/dl) 

**Heart echo**: interventricular septum and posterior wall hypertrophy (13,3mm , 12mm), thickening of the aortic cusps with calcifications, moderate decreased opening, mild aortic insufficiency.

**Figure 4:**
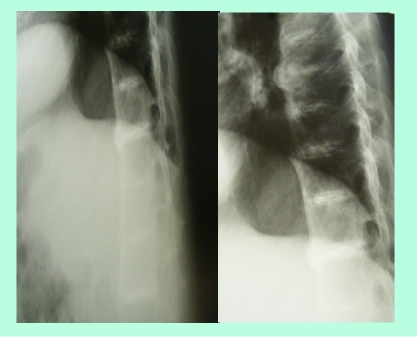
Thoracic spine examination : intervertebral discs calcifications on multiple levels, anterior ligament calcification, disc space narrowing

**Figure 5:**
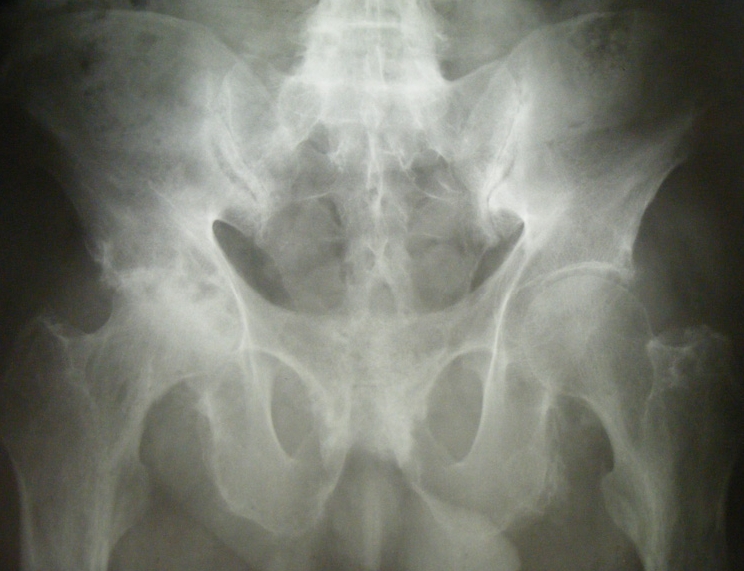
Right hip osteonecrosis stage 4, erosions of pubic symphisis with pseudowidening , entesitis of the ischial tuberosities, narrowing of the left sacroiliac joint

**Figure 6:**
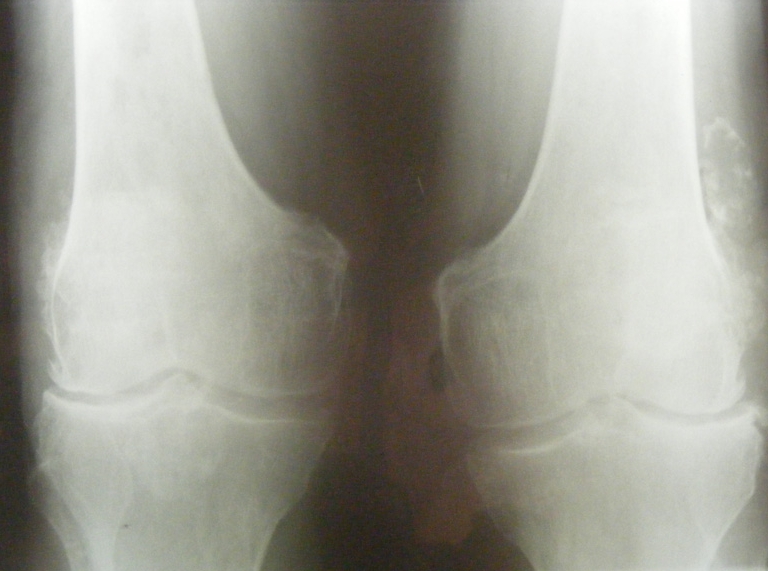
Secondary gonarthrosis, intra–and periarticular calcifications

**Imaging–conventional radiograph **: intervertebral discs calcifications on multiple levels, anterior ligament calcification, disc space narrowing (Figure 4), right hip osteonecrosis stage 4, erosions of pubic symphisis with pseudowidening, entesitis of the ischial tuberosities, narrowing of the left sacroiliac joint(Figure 5), secondary gonarthrosis, intra– and periarticular calcifications (Figure 6)

The patient had all the clinical criteria for  ankylosing spondilitis; but  the sacroiliac Xray changes were quite difficult to score : the incidence was not a good one, the pelvis was a little bit  rotated; however ,narrowing and osteosclerosis of the left sacroiliac joint is visible (although after 30 years of evolution of ankylosin spondylitis we would have expected a stage 4). But the calcifications of the intervertebral discs, history of  kidney stones, aortic stenosis, pigmentations of the sclera, pinna, hands pointed to ochronosis (alkaptonuria). 

Alkaptonuria is a rare autosomal recessive disorder of metabolism caused by deficiency of homogentisic acid oxidase and resulting in accumulation of homogentisic acid in collagenous structures. This causes the classic clinical triad: (1) homogentisic aciduria which presents at birth (pathognomonic sign: urine blackens on standing when oxidized or alkalinized); (2) gradual development of ochronosis after 20 to 30 years of age (deposition of polymers of oxidized homogentisic acid in connective tissues leads to intensive eumelanin–like pigmentation of skin, sclera, cartilages, etc); and (3) degenerative ochronic arthropathies usually in the fourth decade of life. Other important but more rare consequences of alkaptonuric ochronosis are cardiovascular and urinary tract involvement (kidney stones)[1,2,10].Exogenous ochronosis is clinically and histologically similar to its endogenous counterpart; however, it exhibits no systemic effects and is not an inherited disorder[8]. The associated ochronotic discoloration most commonly results from use of products containing hydroquinone (skin lightner). It also occurs following use of antimalarials and products containing resorcinol, phenol, mercury or picric acid[9].

**Figure 7:**
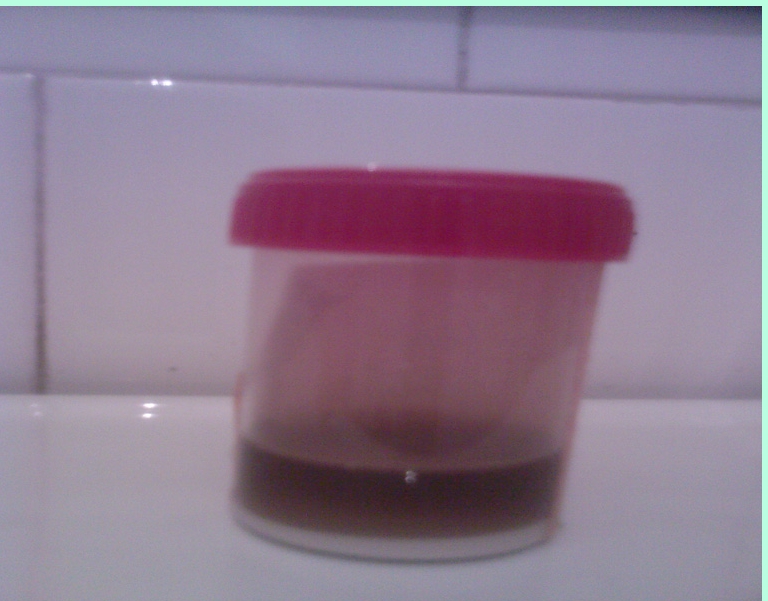


Regarding homogentisic aciduria our patient did not report spontaneous  darkening of the urine , but when left standing for 24 hours the urine turned black. (Figure 7).  Homogentizic aciduria –  Dark staining of the diapers sometimes can indicate the disease in infants (black diapers disease) but only 21% of patients are diagnosed before 1 year of age[1,2];  other simple urinary studies include darkening of urine on standing or  with the addition of sodium hydroxide, black reaction with FeCl_3_.

**Ochronotic discoloration** is most commonly seen in the ear cartilage (pinna) which become thickened and in later stages gross calcification can be seen; ear wax is reddish brown or jet–black. Cutaneous pigmentation can also be detected on the nasal tip, extensor tendons of hands (which appear as a coal black–like tattoo work, may be associated with pitting and hyperpigemented plaques with adherent scales), cheeks, axilla fingernails and buccal mucosa[1,5,11]. Grey–brown or blue black pigmentation of the sclera (often confused with choroidal melanoma) can also be seen[1,12]. The skin markers in our case were  the extensive, progressive pigmentation of the fingers, sclera and auricular cartilages with thickening  and calcifications. The cardiovascular involvement in alkaptonuria is difficult to assesss. The sclerotic change in the cusps, and shrinkage of the non–coronary cusp (due to ochronotic deposits) , appeared to be a stimulus for dystrophic calcification , implying that cardiovascular ochronosis may cause aortic valve regurgitation or stenosis. Mitral and tricuspid valve do not seem to cause increased prevalence of valvular disease. Coronary artheriosclerosis is not increased,[13] When we first saw the patient he had moderate aortic stenosis and insufficiency and he already had surgery for nephrolithiasis (probably related to ochronosis).   Six months after our diagnosis was made  he had aortic valve replacement for severe aortic disease ( class 4 dyspneea) , but unfortunately we do not have the biopsy specimens.

**Ochronotic Arthropathy**–developed in 50% of patients usually after 40 years.In the joints ochronotic pigment is characteristically deposited in the deeper layers of the articular  cartilage, being most pronounced in old cartilage with poor metabolism . The cartilage loses its elasticity, becomes brittle and  stands mechanical strain poorly. The cartilage may thus crack and parts may become separated from the underlying bone  to form loose bodies. Small flakes of cartilage become adherent to the synovial membrane causing  thickening, fibrosis and chondromatosis. At the reflection of the synovial membrane marginal osteophytes form Eventually joint irregularity and exposure of eburnated bone constitute secondary osteoarthritis. Cyst formation and small bone infarctions may occur adjacent to the joint. The cartilage may disappear completely, so that there is spontaneous bony ankylosis. It has been reported that fragments of pigmented articular cartilage may be forced into adjacent cancellous bone and marrow spaces, becoming surrounded by granulation tissue. Conversely, subchondral bone marrow has been reported as invading the affected articular cartilage. The menisci may become pigmented and secondarily calcified In the vertebral column, intervertebral discs become pigmented and secondarily calcified,evoking marginal osteophytosis of the vertebral bodies. In severe cases the vertebral column may become rigid, superficially resembling the bamboo spine of ankylosing spondylitis. The ligaments of the spinal column and its joints may similarly be affected,[1,10].  In ochronosis, the changes in the bone are thought to be less severe than those in cartilage1[4,15]. The accumulation of oxidized and polymerized products of homogentisic acid reduces the cross–linkage of collagen fibers leading to connective tissue failure, cartilage erosion, and progressive degenerative changes[10,15].It is suggested that the detrimental effects of ochronotic pigment on the fibrils of soft connective tissues are avoided by the collagen fibrils of the bones because they are encrusted by a mineral substance and because the newly formed osteoid matrix remains uncalcified for too short of a time to be modified by the pigment. In an ochronotic femoral head, the pigment was not found in osteoblasts but was present in the calcified matrix as well as in the cytoplasmic vacuoles of osteoclasts and in osteocytes, some of which were degenerate or dead.[14] In a series of ochronotic patients, the biochemical markers of bone turnover showed increased bone resorption (high urinary excretion of crosslinked N–telopeptides of type 1 collagen) with an almost normal bone formation in 6 out of 7 patients indicating accelerated bone loss.[15] Importantly, these changes were associated with reduced femoral bone mineral density. While femoral neck bone mineral density was markedly reduced, the lumbar spine bone mineral density was normal or increased. This seeming paradox might be due to extensive intervertebral disc calcification. The first symptoms are usually referred to the spine, the patient complaining of stiffness in the lumbo–sacral region associated with some pain, usually not severe. There may be a gradual extension of the stiffening process throughout the spine,at first with flattening of the lumbar and cervical lordosis, and later with development of kyphosis. There may be a localised scoliosis or lordosis. The rate of involvement of the whole spine depends on the severity of the disease, but it may be complete within ten years. Although there may be forward protrusion of the head as a result of neck deformity, movements of the cervical spine usually remain free. Associated with the spinal changes there is a diminution of stature. Stiffness may become severe, suggesting advanced ankylosing spondylitis . Chest expansion may be reduced because of involvement of costal cartilage and costovertebral joints.[1] Peripheral arthropathy usually is affecting large weight bearing joints and later the shoulders. The knees usually become troublesome at a somewhat later stage than the back. The first symptoms and signs are those of uncomplicated osteoarthritis with pain and swelling. One–third of the patients have an effusion. The knees gradually become stiff and may become deformed, usually in flexion and valgus. The knee may be locked by cartilaginous loose bodies. In the advanced stages of the disease shoulder stiffness and pain may develop. Hip involvement is an uncommon feature[1,10, 16,17,26,27] 

Radiographic changes may precede the onset of symptoms. In the spine the first changes are narrowing of disc spaces, commencing in the thoraco–lumbar region. The vertebral surfaces of the discs become increasingly radiopaque due to calcification, giving a ‘doubling’ of the outline. As the condition progresses the intervertebral discs also become radiopaque. The nucleus pulposus is the last part of the disc to become calcified. The vertebral bodies become porotic, later they may become deformed by compression. Massive osteophyte formation develops at the vertebral margins and intervertebral bony bridging may occur. Cystic changes may be seen in the vertebral bodies. Many of these changes may be interpreted as those of ankylosing spondylitis, but the striking density of the intervertebral discs combined with the usual almost complete absence of ligamentous ossification differentiates the conditions. Kyphotic and scoliotic deformity may be seen involving a limited number of vertebral bodies[10,26,27].

In the knees the first changes are those of calcification of the menisci. There is increased density in the subchondral region with scattered areas of osteoporosis. The articular surfaces become irregular, the joint space becomes narrowed asymmetrically and marginal osteophytes develop. Calcified loose bodies may be seen. Cysts may occur in the bone ends, particularly at the attachments of the ligaments and tendons, and sclerotic islands suggestive of avascular necrosis may be present. In the hips and shoulders similar changes occur with flattening of the femoral and humeral heads[10,26,27].

The articular problems in our case began when he was around 25 years old with lombar back pain not very severe , then he developed gradual extension of the stiffening process throughout the spine; after he turned 50 he noticed mechanical pain  in the right hip and then the  knees. At the time we first saw him he had advanced spondyloarthropathy with diffuse calcifications of the intervertebral discs , severed reduced mobility of the spine , right hip osteonecrosis, knee degenerative arthritis with periarticular calcifications . He also developed visceral involvement. 

**Due to the right hip osteonecrosis he  was refered to a orthopedy clinic for right hip prothesis** .  The approach was a Hardinge lateral direct one. Opening  the articular capsule an exuberant synovitis appeared; it had a vilonodular aspect, with some deep blue coloration in it. After the complete capsular resection and synovectomy, the femoral head was dislocated. At this time a complete blue ring was seen in the subcapital area; same aspect could be observed on the the labrum, with a tendency to extension towards the peripheral acetabular cartilage . At the resection of the femoral head, subchondral osteosclerosis together with cysts were seen (consistent with femoral head osteonecrosis) – 8. When starting the acetabular reaming  poor acetabular bone quality was noticed , with hard bone and cysts 3–4mm deep. An uncemented cup was safely inserted. The femoral stem was inserted in a regular fashion and the prosthesis was reduced.

Cartilage and tendon  biopsy samples with hematoxylin and eosin staining reveal yellowish brown pigment deposits. The deposits do not lose their pigmentation after 3 days in 10% H_2_O_2_

**Figure 8:**
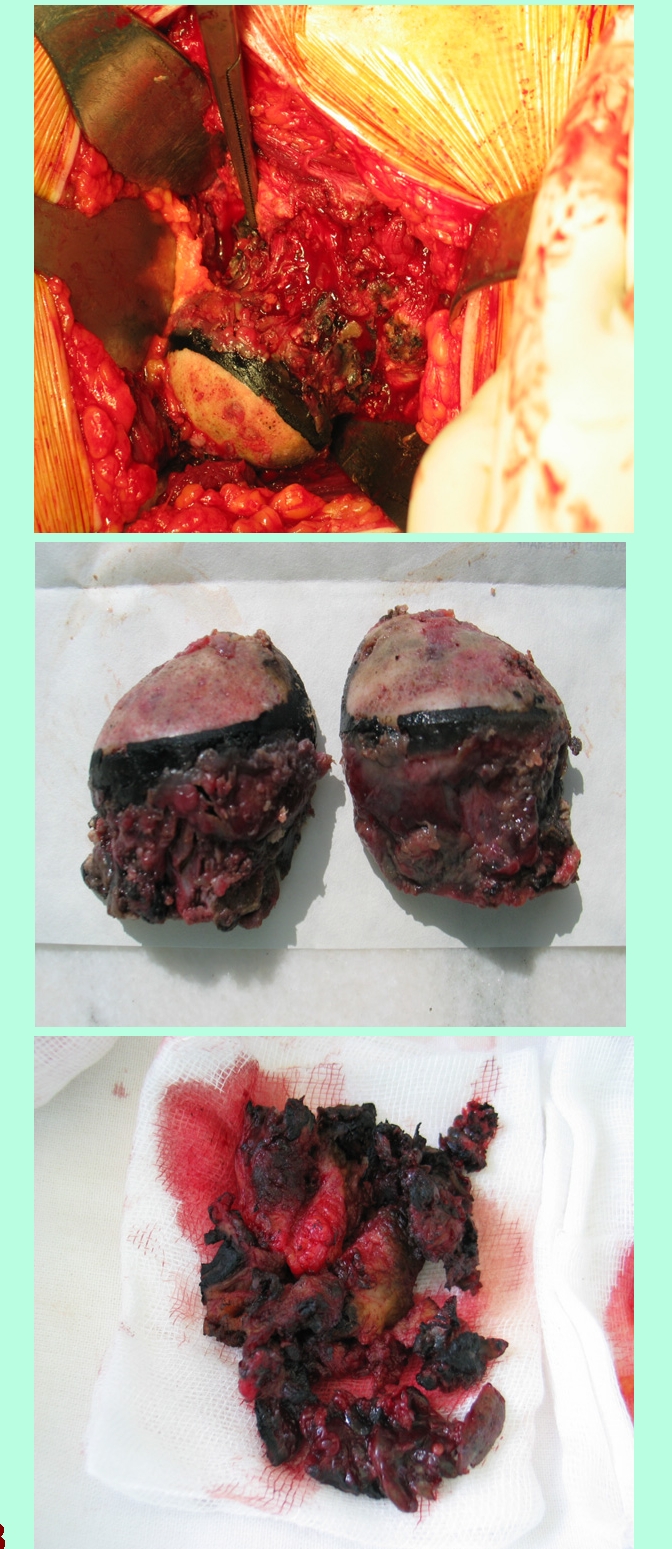


**Figure 9:**
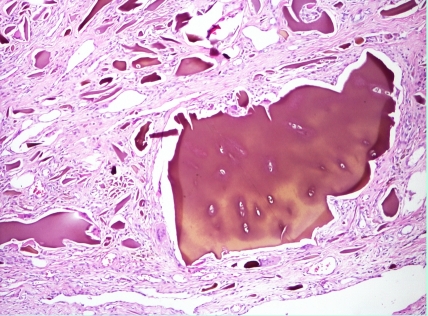


**Figure 10:**
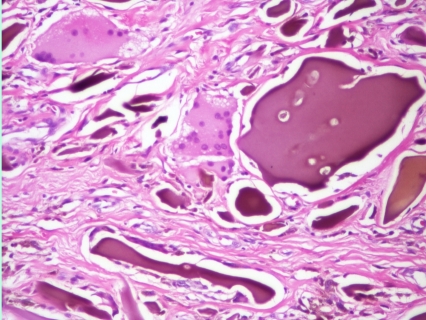
Histology  of hip cartilage – haematoxylin–eosin staining, (magnification X40, X100)

**Figure 10:**
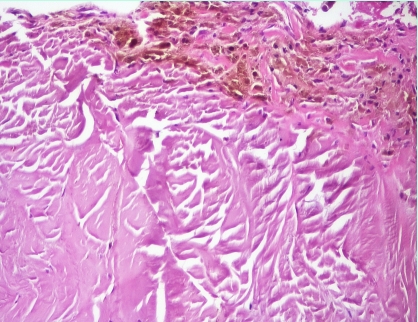
Histology of tendon – Haematoxylin –eosin staining (magnification X40)

## Positive diagnosis 

It was clear enough that our patient had alkaptonuria with multiple visceral involvement : skin (fingers, ear sclera) , severe spondylarthropaty , osteoarthritis of both knees , right hip ostonecrosis , cardiovascular involvement (severe  stenosis and  insufficiency of aortic valve that needed replacement) and urinary tract involvement (nephrolitiasis).  As we could not determine the level of homogentizic acid, the biopsy specimens were our confirmation for a positive diagnosis 

## Differential diagnosis 

The positive diagnosis is quite difficult because ochronotic arthropathy may resemble osteoarthritis, ankylosing spondylitis and Paget[10] .

In **osteoarthritis** the spinal involvement is greatest in the lumbar and cervical regions. Disc calcification is unusual. Progressive stiffening of the whole spine does not occur, and restriction of movement in the cervical spine is less pronounced in osteoarthritis. In the peripheral joints differentiation may be more difficult but calcification of the menisci usually gives a clue to the diagnosis. Periarticular calcification may be a distinguishing feature in ochronotic arthritis[10].

In **ankylosing spondylitis** the pain is more severe, cervical rigidity is more marked and the ESR  is typically raised. Radiographically the intervertebral discs are not normally heavily calcified and there is widespread ossification of the spinal ligaments.In ochronosis bamboo spine, annular ossification, syndesmophytes, erosion, and fusion of sacroiliac joints do not occur. It was claimed that arthropathy, especially axial involvement, is more severe in HLA–B27–positive individuals.[20,21,22] Coexistence of ochronosis and rheumatoid arthritis[18,19] ankylosing spondylitis,[20,21,22] or chondrocalcinosis[23] has also been reported.

**Paget's disease** has only a superficial resemblance and is readily distinguished radiologically and clinically. In it the serum alkaline phosphatase is usually raised[10]. In our case the main question was ‘is it only ochronotic spondylarthropaty or an overlap between alkaptonuria and ankylosing spondylitis?’. The patient's mother was dead,  so we could not  check if she suffered from  ankylosing spondylitis or ochronotic spondylarthropathy.He has only one son with no medical problems. Alkaptonuria is characterized by a remarkable allelic heterogeneity. Affected persons are either homozygous or compound heterozygous for loss of function mutation(s) in HGO.  The family history is important but not conclusive for both diseases. Unfortunatelly at that moment we could not determine  the HLAB27, the genetic marker of ankylosing spondylitis. Our patient'spine Xray showed also anterior longitudinal ligament calcification (rarely described in ochronosis) and severed decreased mobility of cervical (not very usual in alkaptonuria). The left sacroiliac joint was quite narrowed with some osteosclerosis , although after 30 years of ankylosing spondylitis we would have expected a stage 4 sacroiliitis . An  MRI (no longer available due to hip and aortic valve prosthesis) or a CT examination of the sacroiliac joints would have been useful. However at this moment we think that he was either HLAB27positive or had an overlap between alkaptonuria and ankylosing spondylitis.

## Treatment 

Currently there is no specific and effective treatment for alkaptonuria. Although some advocate dietary protein restriction (mainly phenylalanine and tyrosine), and ascorbic acid to reduce urinary homogentisic acid excretion and possibly reverse bone abnormalities[25,26] these observations have not been confirmed in other studies1,[25,26]. 

A direct pharmacologic reduction of homogentisic acid production could be achieved with nitisinone therapy. Nitisinone is a triketone herbicide and potent inhibitor of 4–ydroxy–phenylpyruvate dioxygenase which is responsible for catalyzing the formation of homogentisic acid from hydroxyphenylpyruvic acid. Nitisinone reduced urinary homogentisic acid excretion by approximately 70% in two patients with alkaptonuria. Long-term side effects of nitisinone therapy are under consideration.[1,2] Folic acid, vitamin B12 , ACTH, cortisone and co!chicine have been tried without effect, although some patients have symptomatically been improved for a time by ACTH and cortisone[10].

Methyl thiouracil has been claimed to reduce homogentisic acid formation but there is no conclusive evidence as to its effectiveness[10] 

Our patient received Vitamine C 1g/day, NSAIDs as needed, anticoagulants after valve replacement, betablockers for hypertension and statins. 

Understanding the genetic and molecular basis of alkaptonuria has the potential to offer a new therapeutic approach, enzyme replacement therapy with recombinant HGO. However, despite the theoretical advantage, such a strategy may be difficult to employ. Moreover, it is not known whether accumulation of toxic metabolites of tyrosine will occur, thus excluding this as an acceptable alternative therapy.   In conclusion, diagnosis and management of patients with alkaptonuric ochronosis, a rare inherited disorder, is complex. Advances in orthopaedic and cardiac surgery have enabled many patients to overcome progressive disability. Physicians and surgeons should be aware of multiple system involvement in this disorder, as early recognition and appropriate treatment may significantly improve the quality of life in these patient.

## References

[1] Fischer AA (2004). Alkaptonuric ochronosisi with aortic valve and joint replacement and hip fracture. Clinical medicine and research.

[2] Phornphutkul C (2002). Natural history of alkaptonuria. N Engl J Med.

[3] Stenn FF (1977). Biochemical identification of homogentisic acid pigment in an ochronotic egyptian mummy. Science.

[4] Lee SL, Stenn FF (1978). Characterization of mummy bone ochronotic pigment. JAMA.

[5] Vijaikumar M (2000). Alkaptonuric ochronosis presenting as palmoplantar pigmentation. Clin Exp Dermatol.

[6] Fernandez–Canon  JM (1996). The molecular basis of alkaptonuria. Nat Genet.

[7] Rodriguez JM (2000). Structural and functional analysis of mutations in alkaptonuria. Hum Mol Genet.

[8] Bongiorno MR (2005). Exogenous ochronosis and striae atrophicae following the use of bleaching creams. Int J Dermatol..

[9] Brogeras M (2006). Exogenous ochronosis. Journal of Drugs in Dermatology.

[10] Laskar TH (1970). Ochronotic arthropathy– review of four cases. The Journal of Bone and Joint surgery.

[11] Jebaraj I (2005). Cutaneous markers in Ochronosis. Indian Journal of  medical science.

[12] Felbor U (1999). Ocular ochronosis in alkaptonuria patients carrying mutations in the homogentisate 1,2–dioxygenase gene. Br J Ophthalmol.

[13] Erek E (2004). Cardiac Ochronosis– Valvular Heart Disease with Dark Green Discoloration of the Leaflets. Texas Heart Institut Journal.

[14] Di Franco M (2000). A morphological study of bone and articular cartilage in ochronosis. Virchows Arch.

[15] Aliberti G (2003). Bone metabolism in ochronotic patients. J Intern Med.

[16] Borman P (2002). Ochronotic arthropathy. Rheumatol Int.

[17] Kabasakal Y (1995). Spinal abnormalities similar to ankylosing spondylitis in a 58–year–old woman with ochronosis. Clin Rheumatol.

[18] Kihara T (1994). Coexistence of ochronosis and rheumatoid arthritis. Clin Rheumatol.

[19] Simianer S (1998). Concomitant manifestation of ochronosis and chronic polyarthritis in a patient. Z Rheumatol.

[20] Gemignani G (1990). Coexistence of ochronosis and ankylosing spondylitis. J Rheumatol.

[21] Yagan R (1991). The coexistence of ochronosis and ankylosing spondylitis. J Rheumatol.

[22] Weinberger KA (1991). The coexistence of ochronosis and ankylosing spondylitis. J Rheumatol.

[23] Roth A (1999). A case report: ochronosis in combination with chondrocalcinosis. Z Orthop Ihre Grenzgeb.

[24] Morava E (2003). Reversal of clinical symptoms and radiographic abnormalities with protein restriction and ascorbic acid in alkaptonuria. Ann Clin Biochem.

[25] Forslind K (1988). Alkaptonuria and ochronosis in three siblings. Ascorbic acid treatment monitored by urinary HGA excretion. Clin Exp Rheumatol.

[26] Hamdi N (1999). Ochronotic arthropathy: case report and review of the literature. Int Orthop.

[27] Borman P (2002). Ochronotic arthropathy. Rheumatol Int.

